# Association of Ghrelin Gene Polymorphisms with Fattening Traits and Feed Intake in Pig: A Preliminary Study

**DOI:** 10.3390/ani9070410

**Published:** 2019-07-01

**Authors:** Mirosław Tyra, Katarzyna Ropka-Molik, Katarzyna Piórkowska, Maria Oczkowicz, Magdalena Szyndler-Nędza, Martyna Małopolska

**Affiliations:** 1 Department of Pig Breeding, National Research Institute of Animal Production, Krakowska 1, 32-083 Balice, Poland; 2 Department of Animal Molecular Biology, National Research Institute of Animal Production, Krakowska 1, 32-083 Balice, Poland

**Keywords:** GHRL, fattening performance, pig, feed intake, polymorphism

## Abstract

**Simple Summary:**

From a production point of view, feed intake, growth and carcass quality are among the most important traits in pig breeding. Understanding the mechanisms and processes going on inside the animal’s body can help in the selection of herds and improvement in economic effectiveness. Previous research conducted on different species has showed that ghrelin (GHRL) is responsible for feed intake, efficiency of growth, etc. Thus, evaluation of the regulatory regions and coding sequence of the porcine GHRL gene may be useful as a molecular marker for selected fattening and feed efficiency traits. In this paper, a promising mutation at the locus g.4486C>T was found, which was associated with total feed intake.

**Abstract:**

Numerous studies have been conducted to explain the biological functions and mechanism of ghrelin (GHRL) action in animals. However, the exact role of ghrelin in the regulation of growth and development in pigs is still unclear. The ghrelin gene is considered to be a good candidate marker for the identification of economically important traits in pig production such as feed intake, growth or carcass quality. The objectives of the present study were to investigate the regulatory regions and coding sequence of the porcine GHRL gene and determine the effect of polymorphisms at the ghrelin gene locus on selected fattening traits. Data were obtained from 346 gilts (pure breeds: Landrace, 188; Duroc, 74; Pietrain, 84). The PCR-RFLP (Polymerase Chain Reaction-Restriction Fragment Length Polymorphism) method was used to detect polymorphisms within GHRL. Three polymorphisms were found, one in the promoter region (c.-93A>G) and two in the 3’UTR sequence (g.4428T>C; g.4486C>T). A significant (*p* ≤ 0.01) additive effect on daily gain (negative) and age at slaughter (positive) was obtained at the locus c.-93A>G. However, the most promising mutation was at the locus g.4486C > T, which is associated with total feed intake. Overall, the described GHRL polymorphisms may be useful as molecular markers in pig selection but future studies are required.

## 1. Introduction

The central melanocortin system appears to be a fundamental neuronal pathway that integrates a wide range of signals involved in the regulation of energy homeostasis. In mammals, it is defined as a collection of central nervous system circuits that include arcuate nucleus neuropeptide Y and agouti gene-related protein (NPY/AgRP) neurons or arcuate nucleus proopiomelanocortin (POMC) neurons; brainstem neurons expressing POMC; and downstream targets of these POMC and AgRP neurons expressing the central melanocortin-3 (MC3R) and melanocortin-4 receptors (MC4-R) [1,2].

Leptin, insulin and ghrelin (GHRL) play an important role in the regulation of energy metabolism due to their involvement in the hypothalamic melanocortin pathway [3]. Leptin, as well as insulin, decreases the expression of mRNA for arcuate NPY and AgRP genes, while ghrelin promotes their transcription [4]. Both NPY and AgRP are neuropeptides that are considered to increase food intake and body weight [5,6,7]. Thus, ghrelin stimulates food intake according to its orexigenic effect by activation of NPY/AgRP neurons, and leptin, which demonstrates anorexigenic activity, inhibits it [8]. The relationship that occurs between ghrelin and leptin in body weight regulation has been described as the ghrelin-leptin tango [9].

Ghrelin was discovered in 1999 as an endogenous ligand of the growth hormone secretagogue receptor (GHS-R) and it was purified for the first time from rat stomach [10]. Ghrelin is also secreted from the pituitary gland, lung, kidneys, placenta, ovary, and testes, although in smaller quantities [11]. Porcine GHRL genes has been mapped to SSC13 and located near a QTL associated with average daily weight gain [12]. In human, it has been established that the plasma ghrelin level rise shortly before every meal and falls within one to two hours after the start of eating [13]. Ghrelin has been implicated to both short-term and long-term appetite and body weight regulation. It is assumed that ghrelin plays an important role in feed efficiency, because it is used to maintain energy balance [14]. Furthermore, the potential association between polymorphisms within the GHRL locus and traits of economic importance have been investigated (e.g., feed intake, feed-gain ratio, body weight, efficiency of growth) in different species of animals including beef cattle [15,16,17] and chicken [18,19,20]. In pigs, the role of ghrelin in the regulation of feed intake and daily gain remains unclear. 

In pig production, feed represents 60–70% of total costs [21]. As a consequence, fattening and feed efficiency have a significant impact on economic performance. The present study was performed to investigate the regulatory regions and coding sequence of the porcine GHRL gene and determine the effect of polymorphisms at the ghrelin gene locus on selected fattening traits.

## 2. Materials and Methods 

### 2.1. Animals and Feeding

Ethical approval of this research is not imperative because experimental procedures were performed on biological material. The biological material was derived from pigs maintained and slaughtered in the Test Pig Stations of the National Research Institute of Animal Production, and after carcass evaluation, the meat was intended for consumption.

The analyses were performed on 346 gilts representing 3 pure breeds (Landrace, 188; Duroc, 74; Pietrain, 84). Pigs were maintained at the Pig Test Stations of the National Research Institute of Animal Production (Chorzelów and Pawłowice) under the same housing and feeding system (ad libitum), according to the station methodology [22]. The feeding program was based on two compound feeds: the first, was for growers from 30 kg to 80 kg of body weight, who are responsible for high lean meat yield, and the second feed was for finishers weighing from 80 to 100 kg (Table 1).

### 2.2. Performance Test 

Animals were fattened from 30 to 100 (±2.5) kg of body weight. Before slaughter, pigs fasted for 24 h. The following data were collected: date of birth, test start date, test end date, body weight (once a week) and feed intake during the test period. Based on these data, age at slaughter, length of fattening period, test daily gain (from 30 to 100 kg of body weight), daily gain, feed-gain ratio, total feed intake and daily feed intake were calculated.

### 2.3. Genotyping 

The total DNA from whole blood was isolated using the Wizard DNA Purification Kit (Promega, Madison, WI, USA) according to protocol. For all pigs, three polymorphisms within the GHRL gene were detected using the PCR-RFLP method based on reference sequence NM213807.1. All analyzed polymorphisms were previously detected by PCR-SSCP and Sanger sequencing methods, which was described by Ropka-Molik et al. [23]. Analyzed polymorphisms were identified using endonucleases: MwoI (New England BioLabs, Frankfurt, Germany) for c.-93A>G (rs196957643), localized in 5’gene flanking region; BslI (New England BioLabs) for g.4428T>C (rs196958624) and HgaI (New England BioLabs) for g.4486C>T (rs196950724), both detected in 3’UTR region [23]. The PCR was performed using AmpliTaq Gold 360 DNA Polymerase (Thermo Fisher Scientific, Applied Biosystems, Waltham, MA, USA) according to protocol and the details of the obtained DNA fragment after endonuclease digestion are presented in Table 2.

### 2.4. Statistical Analyses

Statistical analyses were performed using the GLM (General Linear Model) procedure of the SAS (SAS Institute, Cary, NC, v.8.02.2001). The LSM (Least Squares Means) method was used to determine the statistical significance between groups. The linear model was: $Y_{ijkl}=\mu +s_{i}+b_{j}+g_{k}+{\left(bg\right)}_{jk}+e_{ijkl}$ where *Y_ijkl_* is the observation, µ is the overall mean; *s_i_* is the fixed effect of the experimental station, *b_j_* is the fixed effect of breed, *g_k_* is the fixed effect of analyzed genotype, (*bg*)*_jk_* is the covariance between genotype and breed (when significant differences occurred), and *e_ijkl_* is the random error. The sire or sow effect were not included in this model, because analyzed animals were not related (we analyzed offspring from 158 boars and 346 sows, so for one boar, 2.19 gilts were used in this study). Additive and dominance effects were estimated for a total of 346 pigs of all breeds. Additive and dominance effects were calculated according to REG_A procedure (SASv. 8.02), where the additive effect was denoted as −1 and 1 for genotypes AA (CC) and GG (TT), respectively, and the dominance effects are represented as −1 for heterozygotes AG (CT) and 1 for both homozygotes. The data are presented as LSM ± SE.

## 3. Results and Discussion

Multiple genes control the physiological regulation of feed intake and growth. One of them is ghrelin, which stimulates the secretion of growth hormone and is involved in the regulation of food intake and energy balance in rodents [24,25,26] and humans [27,28]. In pigs, administration of ghrelin has an effect on the growth hormone secretion but data regarding the mechanisms and regulation of growth and development are still limited [29].

The occurrence of polymorphism provides an opportunity to use marker-assisted selection to improve quantitative traits [15]. Researchers have detected polymorphisms in ghrelin genes which are associated with carcass and meat quality traits in pigs [12,30], growth and feeding traits in chickens [20], growth traits in goats [31], birth weight and body length in cattle [16], and milk fat and protein synthesis in water buffaloes (Bubalus bubalis) [32]. On the other hand, some of the research results have not shown any association with phenotypes, e.g., studies on humans [33], pigs [23], cattle [34], and sheep [35]. In this study, the genotype frequency of three polymorphisms were identified: one in the promoter region (c.-93A>G) and two in the 3’UTR sequence (g.4428T>C and g.4486C>T). A previous study performed on three pig breeds (Pietrain, Landrace and Large White) indicated a lack of linkage disequilibrium between all three SNPs (single nucleotide polymorphisms) [23]. For the polymorphism c.-93A>G localized in the 5’flanking region and identified by MwoI endonuclease, three genotypes (AA, AG, GG) and two alleles were identified. Allele A (82.6%) had the highest frequency in c.-93A>G, while the observed frequency of the AA genotype was 69.0 %. In both polymorphisms in the 3’UTR region g.4428T>C and g.4486C>T, all three genotypes were found. At the g.4428T>C locus, allele C was much more frequent (69.0%) than T (31%), with 49.4 % for CC, 39.3% for CT and 11.3% for TT. Opposite results were identified in g.4486C>T, where the frequency of allele T was 68.9%, and the frequency of genotypes was: CC 12.7%, CT 37.0% and TT 50.3%. Additionally, the distribution of the observed genotypes (g.4486C>T) was different to the expected genotypes (*p* < 0.05). The frequencies of the analyzed genotypes and alleles are presented in Table 3. Genotypes and allelic frequency differ depending on breed, which probably originate from differences in selection for leanness or fat-related traits [36] (Table 4). Moreover, differences in growth and feeding behavior between breeds have been previously observed [37]. As expected, those differences were confirmed in our study. Statistical analysis (Table 5) showed the significant effect of breed on all fattening traits (*p* < 0.01 for test daily gain, daily feed intake and fattening period, for other traits, *p* < 0.05). 

In our analyses, we used the most cost-intensive traits during the fattening period. In pigs, wide variability in those traits occurs and this is caused by many factors, including breed [38]. Additionally, within the breed, some traits are sex-dependent, in favor of boars [39]. Along with genetic improvement, the differences between individuals in fattening traits decrease. What is more, the genotype of modern pigs (in the same sex) shows variability in this area ranging from 8 to 12%. Our results showed variability ranging from 12 to 15% (Table 5), which provided the basis for conducting research on variability within polymorphisms, not only within breeds. The effect of polymorphism on fattening traits was noted in c.-93A>G (daily gain, age at slaughter), g.4428T>C (test daily gain, daily feed intake, fattening period), and g.4486C>T (total feed intake, fattening period). However, significant associations between genotypes and daily gain and feed intake were not detected by Wojtysiak and Kaczor [30]. Wu et al. [40] showed that infusion of exogenous ghrelin increases body weight gain, but ghrelin had no significant influence on the test average daily weight gain and test average daily feed intake of weanling piglets. Changes in the plasma concentration of ghrelin does not influence feeding behavior, but reflects the energy status of pig regardless of the feeding regime [41]. The results of a study conducted on sheep indicated that significant secretion of ghrelin occurs only when restricted feeding is used and does not occur in ad libitum feeding system [42]. Some authors have also suggested that ghrelin secretion may be stimulated by psychological factors. In chickens, injection of ghrelin reduces food intake [43], and has a negative effect on growth [19].

Our results demonstrated statistically significant (*p* ≤ 0.01) additive effects for daily gain (−33.88, that is negative) and on age at slaughter (9.67, that is, positive) for the c.-93A>G polymorphism. Significant results (*p* ≤ 0.05) for an additive positive effect on test daily gain (26.07) and negative on fattening period (−2.28) were obtained in the g.4428T>C polymorphism. Dominance effect was not significant in all analyzed fattening traits (Table 6). Similarly, Costa et al. [44] detected a strong additive effect and no significant dominance effect on growth traits.

The previous study showed that the c.-93A>G mutation located in the predicted promoter region of the GHRL gene affected the expression level of this gene in the stomach (fundus ventriculis) and thus, may be potentially the most interesting in terms of ghrelin function and phenotypic traits [23]. In the present study, the impact of c.-93A>G polymorphism on fattening performance in pigs was established. Furthermore, two other SNPs were also analyzed due to their localization in the 3’UTR region, which can lead to modification of GHRL transcript stabilization and thus in gene expression level. At the locus c.-93A>G, genotype AA showed differences in the daily gain compared to genotypes GG (*p* < 0.01) and AG (*p* < 0.05). The AA animals were characterized by high daily gain (612 ± 5.78 g), consequently, their age at slaughter was the lowest (169 ± 1.53 days). Thus, the GG animals were older at slaughter than AA (*p* < 0.01) and AG (*p* < 0.05) (Table 7) animals. Tanka et al. [36] reported that the CC homozygous tended to have a higher average daily gain than the AA homozygotes in Landrace gilts. Despite this, no association between c.335 A>C genotype and average daily gain was detected [36]. In our research, the associations between analyzed mutation and growth rate or duration of fattening (from 30 to 100 kg body weight) were not noted. Although, there was a positive tendency in test daily gain and age at slaughter, this was not statistically significant. This might suggest that mutation has the strongest impact on daily gain at the early stages of a piglet’s life and decreases with the pig’s age. It should be emphasized that not only the level of hormone, but also its form has an impact on the analyzed traits. Porcine ghrelin has two major molecular forms, acyleted-ghrelin and deacylated ghrelin [10]. Recently, Lents et al. [37] found that the concentration of acyl-ghrelin was negatively correlated with average daily gain (*p* < 0.01), number of meals (*p* < 0.05) and meal length (*p* < 0.05) in finishing pigs. Pigs with higher concentrations of acyl-ghrelin, consumed more meals for a shorter time and grew more slowly than pigs with lower acyl-ghrelin concentration. In addition, the genetic correlation showed that heavier and fatter pigs ate longer (r2g = 0.356, and 0.474, respectively) and spent more time at the feeder (r2g = 0.339 and 0.593, respectively) [45]. In cattle, the amount of acyl-ghrelin was positively associated with average daily body gain and dry matter intake in ad libitum feeding [46]. Thus, the probability of ghrelin involvement in the regulation of feed intake and growth of finishing beef was very high [47]. In the present study, only g.4428T>C polymorphism showed a significant effect (*p* < 0.05) on daily feed intake, test daily gain and the fattening period. The TT homozygotes were characterized by the highest test daily gain (917 ± 10.55 g) and daily feed intake (2.57 ± 0.03 kg), consequently, their fattening period was the shortest (82.3 ± 0.92 days) compared to CC and TC genotypes (Table 8). Similarly, the results obtained for g.4486T>C showed that pigs with the TT genotype had (*p* < 0.05) the lowest total feed intake (208 ± 4.63 kg) and the shortest fattening period (83.3 ± 1.84 days) compared to homozygous CC pigs (217 ± 2.06 kg; 87.4 ± 0.83 days, respectively). Additionally, TT genotypes had a tendency toward higher test daily gain, daily gain and daily feed intake than CC genotype. The CT pigs did not differ significantly in fattening traits from both homozygotes (Table 9). Reynolds et al. [48] suggested that ghrelin may be involved in the regulation of feed intake in pigs. In coding the sequences of porcine GHRL, we found slight differences in feed intake in all genotypes of analyzed polymorphisms. Only one of them (g.4428T>C), showed statistically significant (*p* < 0.05) differences between the CC genotype (2.41kg), the TC (2.58 kg) and TT genotype (2.57 kg).

## 4. Conclusions

From a production efficiency point of view it is important to reduce costs, hence greater daily weight gain and lower feed intake are preferred. Our results provide information about polymorphisms within porcine ghrelin genes and their relationship to fattening performance. To date, information about the possible effects of mutation within the ghrelin gene on the fattening characteristics of pigs is limited. The presented research confirmed the significant effect of selected polymorphisms on different fattening traits. The mutation c.-93A>G showed no effect on daily feed intake, but could be used in selection programs to reduce the length of the fattening period because a twenty day difference in age at slaughter was observed between AA and GG genotype. However, the frequency of genotype AA in the analyzed population was 70%, so its practical use is limited. In turn, polymorphism g.4428T>C in the 3’UTR region positively influenced the last stage of fattening on traits such as: test daily gain, daily feed intake, and fattening period, but was not directly reflected in age at slaughter or in the feed-gain ratio. The most favorable effect on cost reduction can be found in relation to g.4486C>T, where animals with TT genotypes had lower total feed intake (almost 10 kg) compared to those with CC genotypes. This mutation could be a promising molecular marker in pigs. Despite the lack of one marker that affects all fattening traits, our results provide a basis for future studies and open up new possibilities for further investigations of the described polymorphisms.

## Figures and Tables

**Table 1:** Calculated content of energy and protein in the pig diets.

Item		Grower Diet 30–80 kg	Finisher Diet 80–100 kg
Energy (MJ/Kg)	min	13.50	13.00
Crude protein (%)	min-max	17–19	16–18
Digestible protein (%)	min	13.90	12.80

**Table 2:** The details of the PCR-RFLP method used to identify the polymorphisms in the ghrelin (GHRL) locus.

*GHRL* Gene Region	SNP	Primers Sequence	Endonuclease	Obtained Alleles
5’ gene flanking region	c.-93A>G rs196957643	*FCTTGCCACTTCAGCTCCATT* *R CTCCACTCCCTCATCTGCTC*	*Mwo*I	A-343,301,109bp G-301, 195, 148, 109bp
Exon 4 (3’UTR region)	g.4428T>C rs196958624	*FACCAAGCACGTTTCCTGAAG* *RGAAATCTTCCTGTGGGGTGA*	*Bsl*I	T-282bp; C-173, 109bp
	g.4486C>T rs196950724		*Hga*I	C-180, 71, 31bp T-180, 102bp

SNP: single nucleotide polymorphism.

**Table 3:** Frequencies of genotypes and alleles between different SNP for analyzed population (*n* = 346).

SNP	Alleles (%)		Genotype Observed (%)			Genotype Expected (%)			Significance Level	
	*A*	*G*	*AA*	*AG*	*GG*	*AA*	*AG*	*GG*		
c.-93A>G	*82.6*	*17.4*	*69.0*	*27.2*	*3.8*	*68.0*	*29.0*	*3.0*	ns	( χ^2^= 0.8571)
	*C*	*T*	*CC*	*CT*	*TT*	*CC*	*CT*	*TT*		
g.4428T>C	*69.0*	*31.0*	*49.4*	*39.3*	*11.3*	*47.7*	*42.8*	*9.5*	ns	( χ^2^= 2.2125)
	*C*	*T*	*CC*	*CT*	*TT*	*CC*	*CT*	*TT*		
g.4486C>T	*31.1*	*68.9*	*12.7*	*37.0*	*50.3*	*9.7*	*42.8*	*47.5*	*	( χ^2^= 5.8131)

**Table 4:** The frequencies of genotypes (%) between different SNP for analyzed breeds.

SNP	Landrace N = 188			Duroc N = 74			Pietrain N = 84		
	*AA*	*AG*	*GG*	*AA*	*AG*	*GG*	*AA*	*AG*	*GG*
c.-93A>G	*73.84*	*25.58*	*0.58*	*84.51*	*14.08*	*1.41*	*44.26*	*42.84*	*12.90*
	*CC*	*CT*	*TT*	*CC*	*CT*	*TT*	*CC*	*CT*	*TT*
g.4428T>C	*42.55*	*48.40*	*9.05*	*31.08*	*39.19*	*29.73*	*78.57*	*19.05*	*2.38*
	*CC*	*CT*	*TT*	*CC*	*CT*	*TT*	*CC*	*CT*	*TT*
g.4486C>T	*17.09*	*43.67*	*39.24*	*9.72*	*22.22*	*68.06*	*6.41*	*37.18*	*56.41*

**Table 5:** Means ± SD for fattening traits in all analyzed animals (*n* = 346; * *p* < 0.05; ** *p* < 0.01).

Fattening Traits	Mean	± SE	CV	GLM Significance			
				Breed	c.-93A>G	g.4428T>C	g.4486C>T
Test daily gain(g)	892	*7.07*	14.70	**		*	
Daily gain (g)	605	*4.43*	13.60	*	**		
Feed-gain ratio (kg/kg b.w.)	2.85	*0.02*	12.30	*			
Daily feed intake (kg)	2.49	*0.02*	14.40	**		*	
Total feed intake (kg)	210	*1.40*	12.40	*			*
Age at slaughter(days)	171	*1.20*	13.10	*	**		
Fattening period (days)	85.3	*0.63*	13.60	**		*	*

GLM: General Linear Model; b.w: body weight; CV: coefficients of variation.

**Table 6:** Additive and dominance effect (with SE) obtained for the three GRHL polymorphisms.

Fattening Traits	Effect (mean ± SE)					
	*c.-93A>G*		*g.4428T>C*		*g.4486C>T*	
	Additive	Dominance	Additive	Dominance	Additive	Dominance
Test daily gain(g)	−37.34 ± 19.39	−2.73 ± 11.97	26.07 ± 11.59 *	−0.77 ± 8.06	14.36 ± 12.32	6.90 ± 8.91
Daily gain (g)	−33.88 ± 12.1 **	−6.75 ± 7.47	9.23 ± 7.29	−3.23 ± 5.07	1.23 ± 7.42	3.81 ± 5.37
Feed-gain ratio (kg/kg b.w.)	0.07 ± 0.05	−0.01 ± 0.03	−0.04 ± 0.03	−0.02 ± 0.02	−0.03 ± 0.03	0.03 ± 0.02
Daily feed intake (g)	−0.08 ± 0.05	−0.02 ± 0.03	0.06 ± 0.03	−0.02 ± 0.02	0.01 ± 0.03	0.03 ± 0.03
Total feed intake (kg)	−0.13 ± 3.87	−1.40 ± 2.39	−3.22 ± 2.31	−0.46 ± 1.61	−4.34± 2.29	1.32 ± 1.73
Age at slaughter(days)	9.67 ± 3.28 **	2.40 ± 2.03	−2.06 ± 1.97	1.96 ± 1.37	0.41 ± 2.01	−1.24 ± 1.46
Fattening period (days)	3.24 ± 1.72	0.13 ± 1.06	−2.28 ± 1.03 *	0.29 ± 0.71	−2.07 ± 1.08	−0.49 ± 0.78

b.w: body weight, * the means in rows differ significantly, *p* ≤ 0.05; ** the means in rows differ significantly, *p* ≤ 0.01.

**Table 7:** Differences in growth and fattening traits between pigs with different genotypes at the locus c.-93A>G (LSM ± SE).

Traits	c.-93A>G		
	AA	AG	GG
Test daily gain(g)	891 ± 9.22	859 ± 12.55	826 ± 29.1
Daily gain (g)	612 ± 5.78 ^Aa(A)^	592 ± 7.93 ^ABb(AB)^	545 ± 11.66 ^Bab(B)^
Feed-gain ratio (kg/kg b.w.)	2.84 ± 0.02	2.86 ± 0.03	2.85 ± 0.07
Daily feed intake (kg)	2.50 ± 0.02	2.45 ± 0.04	2.33 ± 0.09
Total feed intake (kg)	209 ± 1.82	212 ± 2.66	208 ± 4.46
Age at slaughter(days)	169 ± 1.53 ^A(A)^	174 ± 2.31 ^Aba(AB)^	189 ± 4.32 ^Bb(B)^
Fattening period (days)	84.5 ± 0.80	87.6 ± 1.14	91.0 ± 3.04

b.w: body weight, the means in rows marked with the different superscript letter differ significantly; capital letters denote significance of difference at *p* ≤ 0.01, in brackets—after Bonferroni correction, whereas small letters denote significance of difference at *p* ≤ 0.05, in brackets—after Bonferroni correction.

**Table 8:** Differences in growth and fattening traits between pigs with different genotypes at the locus g.4428T>C (LSM ± SE).

Traits	*g.4428T>C*		
	CC	TC	TT
Test daily gain(g)	865 ± 19.6 ^a(a)^	893 ± 14.03 ^ab(ab)^	917 ± 10.55 ^b(b)^
Daily gain (g)	597 ± 9.52	612 ± 7.61	615 ± 7.12
Feed-gain ratio (kg/kg b.w.)	2.86 ± 0.06	2.86 ± 0.03	2.76 ± 0.03
Daily feed intake (kg)	2.41 ± 0.06 ^a^	2.58 ± 0.03 ^b^	2.57 ± 0.03 ^b^
Total feed intake (kg)	211 ± 4.64	209 ± 2.63	205 ± 2.01
Age at slaughter(days)	174 ± 4.73	168 ± 2.16	170 ± 1.86
Fattening period (days)	87.3 ± 2.04 ^a(a)^	84.0 ± 1.19 ^ab(ab)^	82.3 ± 0.92 ^b(b)^

b.w: body weight, the means in rows marked with the different superscript letters differ significantly; small letters denote significance of difference at *p* ≤ 0.05, in brackets—after Bonferroni correction.

**Table 9:** Differences in growth and fattening traits between pigs with different genotypes at the locus g.4486C>T (LSM ± SE).

Traits	*g.4486C>T*		
	CC	CT	TT
Test daily gain(g)	868 ± 8.93	869 ± 12.34	897 ± 21.99
Daily gain (g)	607 ± 6.05	601 ± 7.22	610 ± 14.08
Feed-gain ratio (kg/kg b.w.)	2.91 ± 0.03	2.87 ± 0.03	2.84 ± 0.05
Daily feed intake (kg)	2.51 ± 0.02	2.47 ± 0.04	2.53 ± 0.05
Total feed intake (kg)	217 ± 2.06 ^a^	210 ± 2.02 ^ab^	208 ± 4.63 ^b^
Age at slaughter(days)	169 ± 1.73	172 ± 1.83	170 ± 3.74
Fattening period (days)	87.4 ± 0.83 ^a(a)^	86.3 ± 1.06 ^ab(ab)^	83.3 ± 1.84 ^b(b)^

b.w: body weight, the means in rows marked with the different superscript letter differ significantly; capital letters denote significance of difference at *p* ≤ 0.01, in brackets—after Bonferroni correction whereas small letters denote significance of difference at *p* ≤ 0.05, in brackets—after Bonferroni correction.
